# Antibiotic‐Induced Dysbiosis of the Gut Microbiota Shifts Host Tryptophan Metabolism and Increases the Susceptibility of Mice to Pulmonary Infection With *Pseudomonas aeruginosa*

**DOI:** 10.1111/imm.13932

**Published:** 2025-05-19

**Authors:** Camila Bernardo de Brito, Raquel Duque do Nascimento Arifa, Rafael de Oliveira Bezerra, Carlos Eduardo Dias Igídio, Bárbara Maria de Amorim‐Santos, Anna Clara Paiva de Menezes Santos, Larissa Mendes Barbosa, João Paulo Pezzini Barbosa, Larissa Marcely Gomes Cassiano, Markus Kohlhoff, Micheli Fagundes, Rafaela Ribeiro Álvares Batista, Celso Martins Queiroz‐Junior, Alessandra M. Saliba, Juliana Divina Almeida Raposo, Fernão Castro Braga, Roney Santos Coimbra, Mauro Martins Teixeira, Caio Tavares Fagundes, Danielle G. Souza

**Affiliations:** ^1^ Laboratório de Interação Microrganismo‐Hospedeiro, Departamento de Microbiologia Instituto de Ciências Biológicas, Universidade Federal de Minas Gerais Belo Horizonte MG Brazil; ^2^ Centro de Pesquisa e Desenvolvimento de Fármacos, Instituto de Ciências Biológicas, Universidade Federal de Minas Gerais Belo Horizonte MG Brazil; ^3^ Instituto René Rachou (IRR) ‐ FIOCRUZ. FIOCRUZ MG Belo Horizonte MG Brazil; ^4^ Departamento de Microbiologia e Imunologia Universidade do Estado do Rio de Janeiro Rio de Janeiro Brazil; ^5^ Departamento de Produtos Farmacêuticos Faculdade de Farmácia, Universidade Federal de Minas Gerais Belo Horizonte MG Brazil

**Keywords:** AHR and *Pseudomonas aeruginosa*, antibiotics, host‐targeted therapy, IDO1, inflammation, neutrophils

## Abstract

*Pseudomonas aeruginosa* is an opportunistic bacterium that mainly infects those who have previously been treated with antibiotics. We hypothesised that antibiotic treatment disrupts tryptophan metabolism, leading to increased susceptibility to *P. aeruginosa* infection. Our results showed that mice receiving antibiotics exhibited intestinal dysbiosis with alterations in host tryptophan metabolism, a higher mortality rate and a higher bacterial load compared to eubiotic mice. In the lungs of the dysbiotic mice, there was an increase in IDO1 (Indoleamine 2,3‐dioxygenase 1) activity and an accumulation of kynurenine after infection, and IDO1^−/−^ mice were resistant to infection after induction of dysbiosis. Importantly, dysbiosis led to increased expression and activation of AHR (Aryl Hydrocarbon Receptor) in an IDO1‐dependent manner. Blocking AHR activation in dysbiotic mice resulted in a lower bacterial load. Our data showed that increased AHR activation by kynurenine was associated with decreased phagocytosis of *P. aeruginosa* by macrophages and neutrophils. In conclusion, our results indicate that dysbiosis resulting from prolonged antimicrobial treatment alters tryptophan metabolism, leading to activation of the IDO1–AHR axis and increasing susceptibility to *P. aeruginosa* infection. Furthermore, these data suggest that IDO1 or AHR are potential host targets for the prevention of opportunistic infections in patients undergoing antimicrobial therapy.

## Graphical abstract

Antibiotic‐induced dysbiosis of the gut microbiota shifts host tryptophan metabolism and impairs the innate immune response during *Pseudomonas aeruginosa* pulmonary infection. (A) Eubiosis: During eubiosis, the intestinal microbiota metabolises tryptophan into indole metabolites, which keeps tryptophan levels within normal serum concentrations. However, during a *Pseudomonas aeruginosa* infection, an inflammatory response induces tryptophan to be converted into kynurenine. This leads to an increase in kynurenine concentration in the lungs. Once *P. aeruginosa* is recognised by resident cells, alveolar macrophages will phagocytose the bacteria, initiating an inflammatory response that results in the massive recruitment of neutrophils. Neutrophils will then phagocytose and eliminate the bacteria, effectively controlling the bacterial load in the lungs. (B) Dysbiosis: The antimicrobial cocktail induces dysbiosis in the intestinal microbiota, decreasing bacterial groups that metabolise tryptophan into indole. This process causes an elevation in serum tryptophan levels. In response to an infectious stimulus, there is an increase in the activity of the enzyme IDO1, which raises kynurenine concentrations in the lungs. The kynurenine activates the AHR receptor, which hinders the phagocytosis of *Pseudomonas aeruginosa* by alveolar macrophages. This impairment in phagocytosis results in a decreased influx of neutrophils. Additionally, the neutrophils that are recruited exhibit increased expression of AHR, which can also be activated by excess kynurenine, further disrupting the ability to phagocytose the bacteria. Created in BioRender. Brito (2025) https://BioRender.com/z10g943.

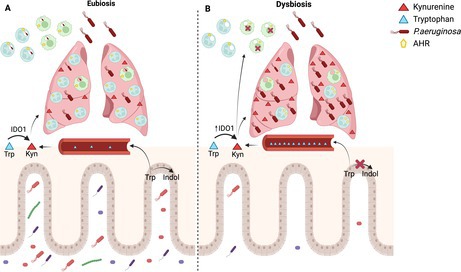

## 1 Introduction

*Pseudomonas aeruginosa* is a Gram‐negative bacillus that may cause diverse clinical diseases in humans, including pneumonia and bloodstream infections. This opportunistic bacterium primarily infects immunocompromised and hospitalised patients who have previously been treated with broad‐spectrum antibiotics. It is a major cause of pneumonia in ventilated patients and has a high mortality rate [1]. Unfortunately, over the years, multidrug‐resistant (MDR) strains of *P. aeruginosa* have become more prevalent, complicating the treatment of infected patients and increasing hospitalisation and mortality rates [1]. As a result, host‐targeted therapy has emerged as a promising strategy to overcome antimicrobial resistance by modifying the host's response to the infection, for example by enhancing the protective immune response or controlling the excessive inflammatory response [2].

The use of antibiotics is one of the main triggers for dysbiosis of the gut microbiota. Their use can alter the microbiota's taxonomic, genomic and functional characteristics and affect its richness and diversity [3]. In contrast to the eubiotic microbiota, which fulfils several important functions for the host, such as the generation and maintenance of an efficient immune response, the maintenance of the intestinal epithelial barrier, the synthesis and absorption of nutrients and metabolites, and protection against pathogens through the secretion of bactericidal substances and niche competition [4, 5, 6, 7, 8, 9, 10, 11], a dysbiotic microbiota is associated with a predisposition to various diseases, including asthma, diabetes and obesity, and leads to greater susceptibility to infections caused by *Clostridium difficile* , *Salmonella enterica* serovar *Typhimurium* and *Citrobacter rodentium* [12, 13]. In addition, prolonged use of antibiotics has been associated with a decrease in products of bacterial metabolism such as short‐chain fatty acids, secondary bile acids and tryptophan metabolites [14].

Tryptophan is an essential amino acid that should be obtained from dietary intakes such as fish, fruit, eggs, meat and milk. In addition to the important function that tryptophan has in protein synthesis, this amino acid is also a precursor of bioactive compounds. To this end, tryptophan is catabolised via two main pathways: the serotonin pathway and the kynurenine pathway. The kynurenine pathway is responsible for the metabolism of about 90% of free tryptophan, where tryptophan is metabolised to kynurenine by TDO (tryptophan‐2,3‐dioxygenase) or by IDO1 (indoleamine‐2,3‐dioxygenase 1). The TDO enzyme is mainly found in liver cells, while IDO1 is found in cells of the nervous system, the immune system and the intestinal epithelium. After kynurenine is formed, it is converted into kynurenic acid or 3‐hydroxykynurenine, which in turn is converted into the cofactor NAD (Nicotinamide Adenine Dinucleotide), picolinic acid or xanthurenic acid [15, 16, 17]. Kynurenine and its metabolites have been shown to play an important role in allergies, autoimmune diseases, psychiatric and neurodegenerative disorders and infectious diseases such as HIV, tuberculosis and chlamydial infections [18, 19, 20]. Interestingly, in addition to the metabolic pathways in the host, tryptophan can also be converted into various indole metabolites by the gut microbiota [21]. Tryptophan metabolites from host metabolism or microbiota metabolism can activate the aryl hydrocarbon receptor (AHR), a transcription factor that is widely expressed in many cell types, including immune cells such as macrophages, neutrophils and B cells, and plays an important role in immunosuppressive responses [22]. It has been demonstrated that activation of the AHR pathway by kynurenine and its metabolites induces a strong immunosuppressive environment, such as inducing the differentiation of CD4+ T cells to an anti‐inflammatory phenotype and increasing IL‐6 production and IL‐22 by T cells [22]. However, it remains unclear how this pathway can affect bacterial infectious diseases.

Thus, this study aims to investigate the effects of gut microbiota dysbiosis on host tryptophan metabolism and the resulting impact on the innate immune response to *P. aeruginosa* infection. Our results show that dysbiosis induced by prolonged antimicrobial therapy disrupts tryptophan metabolism, leading to increased susceptibility to *P. aeruginosa* infection. This increased susceptibility observed in dysbiotic mice correlates with increased IDO1 enzyme activity and AHR activation, which impair phagocytosis of *P. aeruginosa* and promote exacerbation of infection.

## 2 Material and Methods

### 2.1 Animals

C57BL/6 (WT) mice were obtained from the Biotério Central at UFMG. IDO1^−/−^ mice were provided by the Immunopharmacology Laboratory Animal Facility at the Institute of Biological Sciences (UFMG, Belo Horizonte, Brazil). The experiments were previously approved by the animal ethics committees of UFMG (02/2019). All animals were housed under standard conditions in separate cages with a 12 h night/day cycle and free access to commercial chow and water. Our study examined male and female animals, and similar findings were obtained for both sexes (data not shown), but data shown throughout the manuscript are derived only from experiments using 8‐week‐old SPF female mice.

### 2.2 Dysbiosis Protocol

To induce dysbiosis, mice were given a cocktail of ampicillin (1 g/L), vancomycin (500 mg/L), ciprofloxacin (200 mg/L), Imipenem (250 mg/L), metronidazole (1 g/L) and nystatin (120 mg/L) in their drinking water for 14 days. This protocol was adapted from Robak et al. [23]. Microisolators and water bottles containing the antibiotics were replaced twice a week.

### 2.3 Evaluation of Faecal Microbiota Composition

The stools were homogenised in a 0.9% NaCl solution (10% p/v) for the analysis of total aerobic, enterobacteria and lactic acid bacteria. The homogenates were serially diluted and plated on Brain heart infusion (BHI), MacConkey and MRS agar plates. The plates were incubated aerobically at 37°C for 24 h.

### 2.4 Indole Production by Faecal Microbiota

Indole production by the faecal microbiota was evaluated by incubating fecal pellets from eubiotic and dysbiotic mice in Luria‐Bertani (LB) broth for 48 h. After this time, the samples were centrifuged (300 × g for 10 min) and the supernatant was collected and transferred to 96 well plates. Then 30% v/v trichloroacetic acid was added and incubated at 50°C for 30 min under agitation. After 30 min, the plate was centrifuged at 450 × g for 10 min at room temperature. Then Erlich reagent (4‐(dimethylamino)‐benzaldehyde 1.2% w/v in glacial acetic acid‐ Imbralab Brazil) was added and incubated for 10 min at room temperature. The absorbance was measured at a wavelength of 540 nm. An indole standard curve (Sigma‐Aldrich Brazil with 96% purity) was determined according to the same protocol.

### 2.5 *Pseudomonas aeruginosa* Intranasal Infection

For intranasal infection with *Pseudomonas aeruginosa* , mice were anaesthetised with a solution of xylazine (80 mg/kg) and ketamine (20 mg/kg) (i.p.). The mice were then infected intranasally with 10^7^ UFC of the PAO1 strain or with 10^4^ UFC of the PA103 strain. Inoculation was performed at an OD_600nm_ = 0.1, which corresponds to 10^8^ CFU/mL of *P. aeruginosa* . In some experiments, kynurenine (25 mg/kg) or CH223191 (10 mg/kg) (both from Sigma‐Aldrich) was administered i.p. or s.c., respectively, 1 h before infection. In dysbiotic mice, the antibiotic was removed 12 h prior to infection.

### 2.6 Bronchoalveolar Lavage (BAL)

Bronchoalveolar lavage fluid was collected to assess leukocyte influx into the alveolar space. The trachea was exposed and a polyethylene catheter with an outer diameter of 1.7 mm was inserted. Then, 1 mL of phosphate‐buffered saline (PBS) was instilled through the catheter (three times). After staining with Turk's solution, the number of total leukocytes was determined by counting the cells in a modified Neubauer chamber. The neutrophil influx was determined by cytospin slide confection after staining with Panotico Rápido (Laborclin/Brazil).

### 2.7 Bacteria Load on BAL and in the Lungs

The *Pseudomonas aeruginosa* load in the lungs and BAL was quantified. For this purpose, the lungs were homogenised in NaCl 0.9% v/v (10%p/v). Serial dilutions of lung homogenates and BAL (1:10) were then prepared. 10uL of each dilution was plated in triplicate on *Pseudomonas* isolation agar (Acumedia) and incubated at 37°C for 24 h to determine the number of CFU.

### 2.8 MPO Activity Assay

The influx of neutrophils into the lungs was determined indirectly by assaying MPO activity. After infection with *Pseudomonas aeruginosa* , lung samples were collected and frozen in liquid nitrogen. After thawing and processing, the tissue was analysed for MPO activity by measuring the change in OD at 450 nm with tetramethylbenzidine and hydrogen peroxide. The results were expressed as OD at 450 nm.

### 2.9 Flow Cytometry

Lungs were harvested and processed for leukocyte isolation as previously described [24]. Cells from BAL were harvested and counted in Turk's solution, then incubated with Fc‐block (BD‐Biosciences) and stained with a mixture of fluorochrome‐conjugated antibodies from BioLegend (anti‐CD11b‐V500; anti‐CD45‐PerCP/Cy5.5; anti‐F4/80‐PE/Cy7; anti‐CD11c‐BV450; Anti‐Ly6G Alexa488). After washing and permeabilisation, the cells were stained with a purified anti‐Ahr antibody (BioLegend), followed by staining with a goat anti‐rat IgG‐PE antibody. After washing, cells were suspended in PBS and acquired in a BD FACSCanto II flow cytometer (BD Biosciences), using BD FACSDiva software. Analyses were performed using FlowJo software (Tree Star, Ashland, OR). Assessment of ROS production by neutrophils was performed using CellROX Deep Red Reagent (Invitrogen) as previously described [25]. The gating strategy is demonstrated in Figure S1A.

### 2.10 Quantitation of Tryptophan Metabolites

#### 2.10.1 LC–MS/MS Analysis

Analyzes were performed using a Waters ACQUITY UPLC system (Waters, Milford, Massachusetts, USA), consisting of a binary pump, autosampler, in‐line degasser and photodiode array detector (Waters). Data were processed using MassLynx 4.1 software (Waters). Analyses were performed on an Acquity UPLC BEH C18 ethylene bridged hybrid octadecylsilane column (2.1 × 50 mm i.d., 1.7 μm; Waters) coupled to an Acquity UPLC BEH C18 pre‐column (2.1 × 5 mm i.d., 1.7 μm; Waters). The mobile phase consisted of 0.1% formic acid in water (A) and acetonitrile (B) used under the following elution/re‐equilibration conditions: 5% B for 3 min, 5 to 30% B in 6 min, 30 to 99% B in 6 min, remaining in 99% B for 1 min, and returning to the initial condition (5% B) in 1 min, at a flow rate of 0.3 mL/min. A re‐equilibration interval of 3 min was adopted between runs. The column was set to 40°C, and an injection volume of 5 μL was used.

#### 2.10.2 Spectrometric Conditions

Xeco Triple Quadrupole MS mass spectrometer (Waters, Milford, Massachusetts, USA) equipped with an electrospray ionisation (ESI) source, operating in positive and negative ionisation mode, was used for the analysis. The cone gas flow was set to 60 L/h and the desolvation gas flow to 550 L/h at 400°C, while the collision gas (Ar) was used at a flow rate of 0.10 mL/min. The capillary tension was set to 3.5 KV. Data were accomplished in the multiple reaction mode (MRM) using both specific and non‐specific transitions in positive ionisation mode for kynurenine (m/z 209 → 94 Da and 209 → 146 Da, cone voltage 20 V, collision energy 14/18 V), tryptophan (m/z 205 → 146 Da and 205 → 118 Da, cone voltage 20 V, collision energy 18/26 V), indole‐3‐carboxaldehyde (m/z 146 → 118 Da and 146 → 91 Da, cone voltage 25 V, collision energy 14/22 V) and 3‐indoleacetic acid (m/z 176 → 130 Da and 176 → 103 Da, cone voltage 22 V, collision energy 30/30 V), while the negative ionisation mode was selected for indoxyl sulfate (m/z 212 → 80 Da and 212 → 132 Da, cone voltage 34 V, collision energy 22/20 V). Chromatograms and spectra were generated and processed online using Mass LynxTM software (version 4.1, Waters).

#### 2.10.3 Sample Preparation

200 μL of ice‐cold methanol containing 100 ng/mL phenacetin (Sigma‐Aldrich) was added to 50 μL of the sample in a safety flask. The solution was vortexed and centrifuged (15 000 × *g*) at 4°C for 10 min. An aliquot (100 μL) of the supernatant was then added to 10 mg of C18 resin to remove hydrophobic compounds. 50 μL of the supernatant was collected and dried in a vacuum centrifuge concentrator, and the resulting residue was dissolved in 50 μL of water. The standard solutions used to prepare the calibration curves (as described in the analytical method) were processed in a similar manner. All standard and sample solutions were analysed on the same day they were prepared. Metabolic concentrations were normalised to the total weight of the samples. The solutions were filtered through a 0.22 PTFE membrane before injection into the UPLC system.

#### 2.10.4 Analytical Method

Five‐point calibration curves were generated by injecting 10% methanol solutions of the reference compounds at various concentration ranges for Kynurenine (5–1000 ng/mL), tryptophan (100–10.000 ng/mL), indoxyl sulfate (10–1.000 ng/mL), indole‐3‐carboxyaldehyde (2.5–50 ng/mL) and 3‐indoleacetic acid (5–100 ng/mL) in quintuplicate. Curves were generated by plotting the injected concentrations against the peak areas on two separate days using linear regression (Microsoft Excel 2010 software). The data were combined and the resulting calibration curves were found to be linear over the indicated ranges, with *r* ^2^ > 0.999 for all regression equations. Concentrations of metabolites were calculated using MassLynx 4.1 software (Waters) and expressed in ng/mL as mean ± standard deviation (mean ± SD, *n* = 3).

### 2.11 In Vivo Phagocytosis Assay

To evaluate phagocytosis of *Pseudomonas aeruginosa* by alveolar macrophages in vivo, mice were intranasally infected with 10^7^ UFC of PAO1 for 2 h. Then, the mice were euthanised and BAL was performed. The counts of the number of macrophages containing bacteria in the cytoplasm and the number of bacteria in the macrophages were determined from cytospin preparations. A total of 100 macrophages were counted. The phagocytic index was calculated by the following formula: (total number of engulfed cells/total number of macrophages counted) × (number of macrophages with engulfed cells/total number of macrophages counted) × 100.

### 2.12 BMDM Culture

Macrophages were derived from the bone marrow of C57BL/6 mice and IDO1^−/−^ mice. Bone marrow cells were harvested from the femurs and tibias of the animals and cultured in DMEM containing 20% FBS and 30% L929 cell‐conditioned medium (LCCM) as a source of M‐CSF. After 7 days, BMDMs were fully differentiated for further experimental procedures.

### 2.13 Isolation of Human Peripheral Blood Neutrophils

Peripheral blood from healthy donors was used for the isolation of neutrophils using the Histopaque gradient protocol [26]. Donor blood was collected in ethylenediaminetetraacetic acid (EDTA)‐containing tubes. Neutrophils were separated with a double‐density gradient using Histopaque 10 771 and 11 191 (both from Sigma‐Aldrich). After the isolation of polymorphonuclear cells, erythrocytes were removed by hypotonic lysis. Neutrophils were approximately 95% pure, as confirmed by the morphological appearance by light microscopy.

### 2.14 In Vitro Gentamicin Protection Assays

For phagocytosis, BMDMs and neutrophils were incubated with PAO1 at a MOI of ∼10 for 30 min at 37°C, followed by incubation in 100 μg/mL gentamicin for 15 min at 37°C. For intracellular killing assays, a modification of the previous protocol was used: after a 30 min co‐incubation of bacteria, gentamicin was added and incubated for the following 60 min, during which aliquots were analysed for remaining CFU. In all cases, cells were washed with PBS and then lysed in 200 μL of 0.1% Triton X‐100 in PBS. The lysates were plated on *Pseudomonas* isolation agar and incubated overnight at 37°C. The next day, colonies were counted and relative phagocytosis and killing were determined by counting CFU.

### 2.15 Statistical Analysis

The results are expressed as the median. Data were analysed according to distribution and variances, and differences were compared using analysis of variance (ANOVA). A one‐way or two‐way ANOVA was used, followed by aStudent‐Newman‐Keuls post hoc analysis (normal distribution and equal variances) or Sidak's multiple comparisons test. Survival curves were compared using the log‐rank test. Results with a *p* value < 0.05 were considered significantly different. GraphPad Prism 7.01 software (GraphPad) was used for the analysis.

## 3 Results

### 3.1 Antibiotic Treatment Disrupts Tryptophan Metabolism in Mice and Increases Susceptibility to Infection With *P. aeruginosa*

**FIGURE 1:**
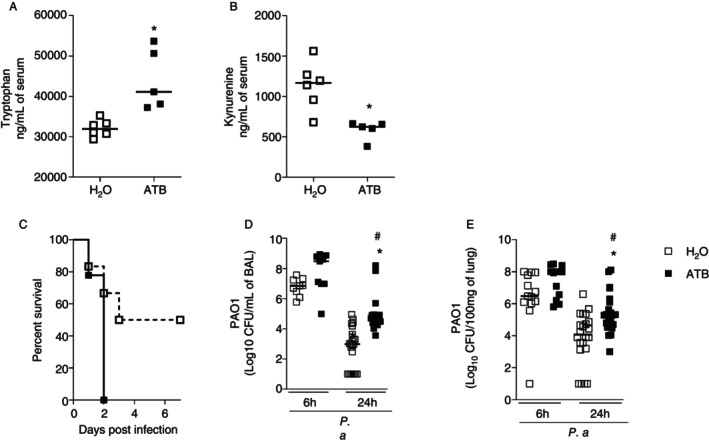
Treatment with an antibiotic cocktail alters host tryptophan metabolism and increases mice susceptibility to *P.aeruginosa* infection. C57/BL6 mice were treated with an antibiotic cocktail in drinking water for 14 days. At the end of the protocol, the mice were euthanized, and serum was collected to determine the concentration of tryptophan (A) and kynurenine (B) by UPLC‐MS. One group of mice was following to determine survival for 7 days (C). In addition, mice were euthanized 6 or 24 h after infection, and BAL and lungs were collected for subsequent analyses: Bacterial load in BAL (D) and lungs (E). In A and B, statistical analyses were performed with the one‐way test ANOVA followed by the Newman–Keuls post‐test. *p < 0.05 versus NI; ^#^ *p* < 0.05 versus H_2_O 24 h. Experimental *N*: 5–8. In C, results are expressed as percentage of survival after infection. Experimental *N* = 6–8. Statistical analysis comparing survival curves was performed with the log‐rank test (Mantel‐Cox). In D and E, the results of 3 independent experiments with experimental *N*: 5–8 were pooled. Statistical analysis was performed with a one‐way test ANOVA followed by a Newman–Keuls post‐test. *p < 0.05 versus NI ^#^ *p* < 0.05 versus H_2_O 24 h.

The initial aim was to develop a model of dysbiosis induced by prolonged treatment with antimicrobials. Mice receiving the cocktail for 14 days showed a reduction in total aerobic bacteria (Figure S1B), lactic acid bacteria (Figure S1C) and enterobacteria (Figure S1D) in the faeces. Mice receiving the antimicrobial cocktail also showed a reduction in indole‐producing bacteria in the faeces (Figure S1E). This change in fecal microbiota was followed by changes in the tryptophan metabolism of the host. The antibiotic‐treated mice showed an increase in tryptophan (Figure 1A) and a decrease in kynurenine (Figure 1B) concentrations in serum. Mice receiving the antimicrobial cocktail also presented a reduction in 16S rRNA gene content in the lung (data not shown).

**FIGURE 2:**
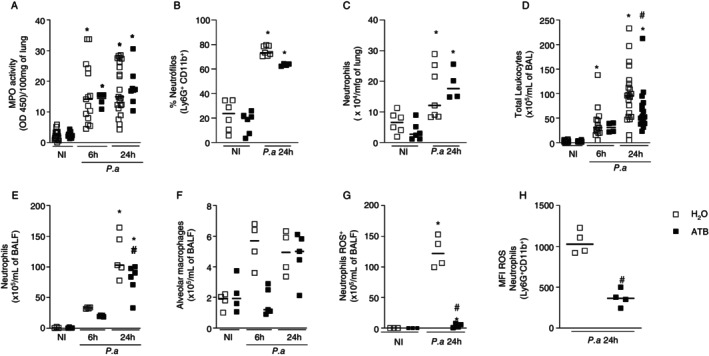
Treatment with an antibiotic cocktail impacts neutrophil influx after infection with *Pseudomonas aeruginosa* . C57/BL6 mice were treated with the antibiotic cocktail for 14 days. At the end of the protocol, mice were intranasally infected with 10^7^ CFU of the PAO1 strain. At 6 and 24 h after infection, mice were euthanized and BAL and lungs were collected for subsequent analyses: MPO activity assay in lung homogenates (A); percentage (B) and the total number of neutrophils (B) by flow cytometry; total leukocytes (C), neutrophil (E), and alveolar macrophage (F) counts in BAL. Production of ROS by neutrophils was determined in alveolar lavage (G, H) by flow cytometry. In A and B, results from three independent experiments were pooled with *N*: 5–8. In C and D, one representative experiment from three independent experiments was shown (*N*: 5). In A–E, statistical analysis was performed with a one‐way test ANOVA followed by a Newman–Keuls post‐test. *p < 0.05 versus N.I. ^#^ *p* < 0.05 versus H_2_O 24 h. In *F*, statistical analysis was performed with Student's *t*‐test. *p < 0.05 versus H_2_O. Experiment *N*: 4.

Next, it was investigated whether pretreatment with antibiotics changes the susceptibility to infection with *P. aeruginosa* . After infection with 10^7^ CFU of *P. aeruginosa* strain PAO1, eubiotic mice showed 50% mortality 6 days post‐infection. Interestingly, dysbiotic mice showed 100% mortality already 2 days after PAO1 infection (Figure 1C). At 6 h post‐infection, the bacterial load in the bronchoalveolar space (Figure 1D) and in the lungs (Figure 1E) was similar in eubiotic and dysbiotic mice. However, 24 h after infection, the bacterial load in the bronchoalveolar space (Figure 1D) and lungs (Figure 1E) was higher in the dysbiotic mice than in the eubiotic mice. Antibiotic treatment also increased susceptibility and higher burden when the mice were infected with the invasive *P. aeruginosa* strain PA103 (Figure S2A–C), suggesting that this phenotype is strain‐independent. To understand if the lung microbiota plays a role in this phenotype, we treated mice with streptomycin, a non‐absorbable antibiotic that has been shown only to affect bacterial load in the gastrointestinal tract without impacting the lung microbiota [27, 28]. Similar to the antibiotic cocktail, streptomycin‐treated mice showed an increase in the *P. aeruginosa* load in both the BAL (Figure 2D) and the lungs (Figure 2E) in comparison to that found in water‐treated mice.

Myeloperoxidase (MPO) activity analysis, an indirect measurement of neutrophil influx, showed no differences between eubiotic and dysbiotic mice after 6 and 24 h of *P. aeruginosa* infection (Figure 2A). A similar result was observed in the percentage and total numbers of neutrophils influx to the lung (Figure 2B,C) upon 24 h of *P. aeruginosa* infection, as assessed by flow cytometry. However, dysbiotic mice showed a lower influx of total leukocytes (Figure 2D) and neutrophils (Figure 2E) into the bronchoalveolar space 24 h after infection than eubiotic‐infected mice. The number of alveolar macrophages in the bronchoalveolar space was not altered after infection (Figure 2F). In addition to the reduced number of neutrophils in the alveoli, neutrophils reaching this compartment in *P. aeruginosa* ‐infected dysbiotic mice exhibited lower levels of reactive oxygen species (ROS) compared to their eubiotic counterparts (Figure 2G,H). Overall, these results suggest that antibiotic treatment led to changes in the gut microbiota and an accumulation of tryptophan in the bloodstream. In addition, antibiotic‐treated mice were more susceptible to infection due to reduced neutrophil mobilisation to and activation in the alveolar space.

### 3.2 IDO1‐Mediated Kynurenine Production Controls Susceptibility to *P. aeruginosa* Infection in Dysbiotic Mice

**FIGURE 3:**
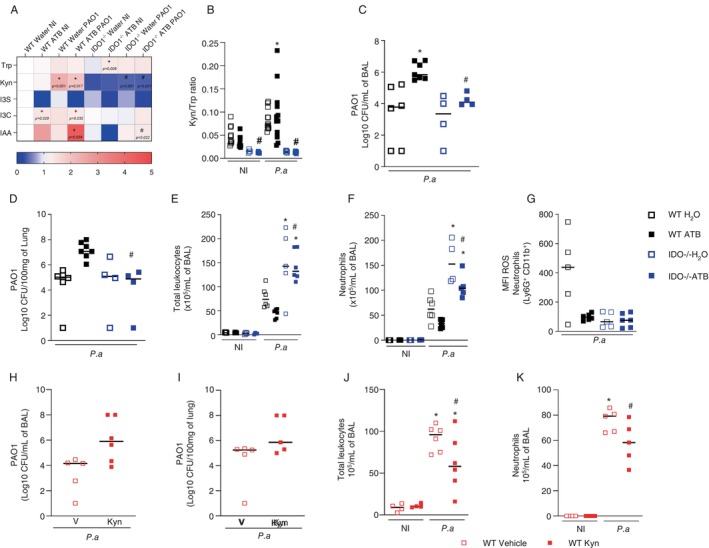
IDO1‐mediated kynurenine production impairs clearance of *P. aeruginosa* during dysbiosis. C57/BL6 and IDO1^−/−^ mice were treated with the antibiotic cocktail for 14 days. At the end of the protocol, the mice were intranasally infected with 10^7^ CFU of the PAO1 strain. Twenty‐four hours after infection, the mice were euthanized, and the lungs were harvested for quantification of Trp metabolites by UPLC‐MS, and the ratio of Kyn to Trp was calculated (A and B). Twenty‐four hours after protocol infection, another group of mice were euthanized and BAL and the lungs were examined for bacterial load in BAL (C) and the lungs (D). The total number of leukocytes (E), neutrophil granulocytes in BAL (F), and the production of ROS by neutrophil granulocytes in BAL (G) were analysed. C57BL/6 mice received 25 mg/kg kynurenine or vehicle intraperitoneally 1 h before intranasal infection with 10^7^ CFU of the PAO1 strain. After 24 h, BAL and lungs were harvested to determine bacterial load (H and I) and total infiltrated leukocyte and neutrophil count at BAL (J and L). In A, results are expressed as relative increase over the mean of the group water NI. Statistical analysis was performed with the two‐way test ANOVA and Sidak multiple comparison test. *p < 0.05 versus respective NI group, #*p* < 0.05 versus respective WT group and +*p* < 0.05 versus respective water‐treated group. In E and F, statistical analysis was performed using the two‐way test ANOVA and the Sidak multiple comparison test. In C and D *p < 0.05 versus H_2_O and #*p* < 0.05 versus WT ATB. In E *p < 0.05 versus NI #*p* < 0.05 versus WT 24 h. In F *p < 0.05 versus H_2_O. In G–J, statistical analysis was performed with a one‐way test ANOVA followed by a Newman–Keuls post‐test. In F and G #*p* < 0.05 versus vehicle. In H and I *p < 0.05 versus NI, #*p* < 0.05 versus vehicle treated infected. Experimental *N*: 4–6.

Infection with *P. aeruginosa* led to an increase in kynurenine concentration in the lungs of both eubiotic and dysbiotic mice compared to the respective NI controls (Figure 3A). In addition, dysbiotic‐infected mice showed an increase in indole‐acetic acid (IAA) in the lungs compared to eubiotic‐infected mice. However, the concentration of indoxyl‐sulphate (I3S) and indole‐carboxyaldehyde (I3C), other tryptophan metabolites, did not change after infection (Figure 3A).

Similar to the WT mice, prolonged treatment with antibiotics also led to dysbiosis in theIDO1^−/−^ mice. These mice showed a decrease in the total number of aerobic bacteria and lactic acid bacteria in the faeces after antibiotic treatment (Figures S3A,B). UPLC‐MS analysis showed that IDO1^−/−^ mice exhibited decreased kynurenine production (Figure 3A). Furthermore, infection with *P. aeruginosa* resulted in a higher Kyn:Trp ratio in the lungs of the dysbiotic WT mice than in the lungs of the eubiotic WT mice (*p* = 0.0004), suggesting that IDO1 may be more active in the former group (Figure 3B). As expected, the Kyn:Trp ratio is very low in both groups of IDO1−/− mice, indicating an important role of this enzyme in the metabolization of tryptophan to kynurenine and in increasing the Kyn:Trp ratio during infection with *P. aeruginosa*. Importantly, dysbiotic IDO1^−/−^ mice had a lower bacterial load in the BAL and lungs compared with dysbiotic WT mice (Figure 3C,D). Interestingly, both infected groups of IDO1^−/−^ mice showed an increase in leukocytes and neutrophil influx into the BAL compared to WT. However, dysbiosis led to a decrease in leukocytes and neutrophil count in IDO1^−/−^ mice compared with eubiotic IDO1^−/−^ mice (Figure 3E,F). Neutrophils from infected IDO1^−/−^ mice showed a decrease in ROS production in both the eubiotic and dysbiotic states, showing that the increase in neutrophil activation is not the reason for less bacteria in IDO1^−/−^ mice (Figure 3G). Interestingly, dysbiosis induced a decrease in inflammatory score in the lung of WT mice but not in IDO1^−/−^ mice (Figure S4A).

Based on these results, the next step was to investigate whether treatment of eubiotic mice with kynurenine alters their susceptibility to infection with *P. aeruginosa* . Treatment of eubiotic WT mice with 25 mg/kg kynurenine resulted in an increased *P. aeruginosa* load in the BAL (Figure 3H). We could not detect any difference in the bacterial load in the lungs of the kynurenine‐treated group compared to the vehicle‐treated mice (Figure 3I). In addition, the number of leukocytes, especially neutrophils, was lower in the BAL of the kynurenine‐treated mice (Figure 3J,K).

**FIGURE 4:**
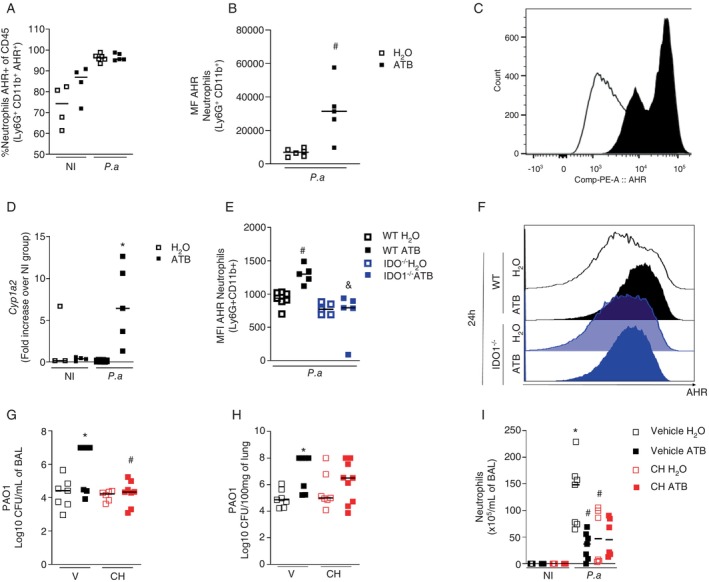
AHR upregulation and activation mediate the increased susceptibility of dysbiotic mice to *P. aeruginosa* infection. C57/BL6 mice were treated with the antibiotic cocktail for 14 days. At the end of the protocol, mice were intranasally infected with 10^7^ CFU of the PAO1 strain. Twenty‐four hours after the infection, mice were euthanized and BAL was harvested for flow cytometric analysis to determine the percentage of neutrophils expressing AHR (A), the MFI of AHR staining in neutrophils (B and C) and lungs were collected to assess *Cyp1a2* expression (D) and MFI of AHR expression in neutrophils (E‐F). A group of C57/BL6 mice treated with an antibiotic cocktail received 10 mg/kg of the AHR antagonist CH223191 or vehicle intraperitoneally 1 h before infection with 10^7^ CFU of the PAO1 strain. After 24 h, mice were euthanized, and BAL and lungs were harvested for quantification of bacterial load (G and H) and total neutrophil count in the BAL (I). In A–D, statistical analysis was performed with the one‐way ANOVA test followed by the Newman–Keuls post‐test. *p < 0.05 versus respective NI; #*p* < 0.05 versus H_2_O 24 h. Experimental *N*: 3–6. In E, statistical analysis was performed with the two‐way ANOVA test followed by Sidak's for multiple comparisons test. #*p* < 0.05 versus H_2_O 24 h; &*p* < 0.05 versus WT ATB 24 h. In G–I, statistical analysis was performed with the two‐way ANOVA test followed by Sidak's multiple comparisons test. *p < 0.05 versus respective N.I.; #*p* < 0.05 versus H_2_O 24 h. Experimental *N*: 5–7.

Given previous findings on the association between elevated IDO1 activity and susceptibility to infection and considering that kynurenine can activate the aryl‐hydrocarbon receptor (AHR) [29], we examined AHR expression in neutrophils and their transcriptional activity in the lung after *P. aeruginosa* infection. Although there were no differences in the percentage of neutrophils expressing AHR between eubiotic and dysbiotic infected mice (Figure 4A), we found that the AHR MFI was greater in neutrophils from dysbiotic mice than in neutrophils from eubiotic mice (Figure 4B,C). In addition, *Cyp1a2* expression is increased in lung tissue from infected dysbiotic mice compared to infected eubiotic mice (Figure 4D). Importantly, the increased AHR MFI after infection in neutrophils from dysbiotic WT mice was not detected in neutrophils from dysbiotic IDO1‐deficient mice (Figure 4E,F).

Because AHR expression was increased in neutrophils from WT mice but not in neutrophils from IDO1^−/−^ mice, we hypothesized that kynurenine‐mediated AHR receptor activation may be involved in the greater susceptibility of dysbiotic mice to pulmonary infection by *P. aeruginosa* . To test this hypothesis, we pretreated WT mice infected with *P. aeruginosa* with an AHR antagonist (CH223191). Our results showed that treatment with CH223191 reversed the dysbiosis‐induced increase in bacterial load in BAL and lungs (Figure 4G,H). Under eubiotic conditions, treatment with CH223191 decreased the number of neutrophils in BAL. However, there was no difference between the dysbiotic group treated with vehicle or CH223191 (Figure 4I). Overall, these results suggest that AHR expression during infection in dysbiotic mice is secondary to IDO1 activity, and its activation is involved in inefficient control of the bacterial load associated with dysbiosis.

### 3.3 Enhanced Kynurenine‐Mediated AHR Activation Interferes With *P. aeruginosa* Phagocytosis

**FIGURE 5:**
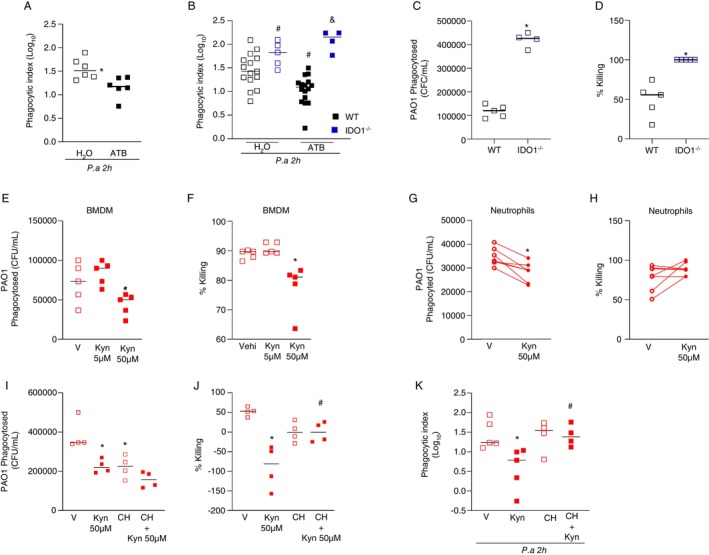
IDO1‐mediated increased kynurenine production disrupts phagocytosis of *P. aeruginosa* in an AHR dependent manner. C57/BL6 and IDO1^−/−^ mice were treated with the antibiotic cocktail for 14 days. At the end of the protocol, mice were intranasally infected with 10^7^ CFU of the PAO1 strain. After 2 h after infection, mice were euthanized, and BAL was collected for determination of phagocytosis index (A and B). Bone marrow cells from WT and IDO1^−/−^ mice were harvested and differentiated into macrophages for 7 days. Phagocytosis and killing assays were then performed to determine the CFU of phagocytosed *P. aeruginosa* (C), and the percentage of killing (D). BMDM cells from WT mice and human peripheral blood neutrophils were treated with kynurenine at concentrations of 5 or 50 μM for 1 h. After 1 h of treatment, phagocytosis and killing assays were performed to determine the CFU of phagocytosed *P. aeruginosa* (E and G) and the percentage of *killing* (F and H). BMDM cells from WT mice were treated with 10uM CH223191 for 1 h and following by treatment with kynurenine at a concentration of 50 μM for 1 h. After the treatments, phagocytosis (I) and killing (J) assays were performed to determine the CFU of phagocytosed *P. aeruginosa* . For in vivo evaluation, C57BL/6 mice received 10 mg/kg of the AHR antagonist CH223191 or vehicle, and after 1 h of the treatment, mice received 25 mg/kg kynurenine or vehicle. Mice were infected intranasally with 10^7^ CFU of the PAO1 strain, and 2 h after infection, mice were euthanized and BAL collected for phagocytosis index (K) determination. In A, C and G statistical analyses were performed using Student's *t*‐test. In B, E, F and K statistical analysis was performed using the one‐way ANOVA test followed by the Newman–Keuls post‐test. In G and H statistical analysis was performed in a paired way. In A *p < 0.05 versus H_2_O. In B #*p* < 0.05 versus WT H_2_O; &*p* < 0.05 versus WT ATB. In C and D *p < 0.05 versus WT. In E–K *p < 0.05 versus vehicle and #*p* < 0.05 versus kynurenine 50 μM. Experimental *N*: 4–13.

Our results suggest that the production of kynurenine from tryptophan by IDO1 inhibits the control of bacterial load in dysbiotic mice in an AHR‐dependent manner. Since the production of ROS was not restored after infection in neutrophils from dysbiotic IDO1^−/−^ mice, we hypothesised that kynurenine might be involved in the control of bacterial uptake. To test this, we performed an in vivo assay of phagocytosis 2 h after infection with *P. aeruginosa* in eubiotic and dysbiotic mice. At this time point, eubiotic and dysbiotic mice had similar numbers of leukocytes in BAL (Figure S4B). However, in WT mice, the state of dysbiosis was associated with a lower phagocyte index than in eubiosis (Figure 5A). Interestingly, IDO1^−/−^ mice showed an increase in phagocyte index in both eubiosis and dysbiosis (Figure 5B). It is worth noting that antibiotic treatment did not alter the leukocyte count, and at this time of infection, all groups had the same number of leukocytes in the alveolar space (data not shown). BMDM from IDO1^−/−^ mice showed increased phagocytosis (Figure 5C) and killing (Figure 5D) of *P. aeruginosa* compared to BMDM from WT mice. The same profile was observed in alveolar macrophages (Figure S4C,D). Treatment of BMDM with 5 μM kynurenine did not affect phagocytosis (Figure 5E) or killing (Figure 5F) of *P. aeruginosa* . However, treatment with 50 μM kynurenine resulted in decreased phagocytosis (Figure 5F) and killing (Figure 5F) of *P. aeruginosa* by BMDMs. Kynurenine treatment did not alter macrophage viability (Figure S4E) or *P. aeruginosa* growth (Figure S4F). Similarly, human peripheral blood neutrophils incubated with 50 μM kynurenine showed a decrease in phagocytosis of *P. aeruginosa* (Figure 5G). Nevertheless, the treatment with kynurenine did not alter the ability of neutrophils to kill *P. aeruginosa* (Figure 5H). Treatment of BMDM cells with 10 μM of the AHR antagonist (CH223191) reduced phagocytosis of *P. aeruginosa* compared with BMDM that received vehicle (Figure 5I). The killing of *P. aeruginosa* was not affected by the AHR antagonist (Figure 5J). Surprisingly, BMDM cells treated with kynurenine after incubation with the AHR antagonist phagocytosed and killed *P. aeruginosa* as efficiently as BMDM that received the antagonist alone (Figure 5I,J). In addition, cells receiving kynurenine after the AHR antagonist were more efficient at killing *P. aeruginosa* compared with BMDM cells receiving kynurenine alone (Figure 5I,J). Finally, macrophages in the BAL of mice treated with kynurenine before infection with *P. aeruginosa* showed a lower phagocytosis index than macrophages from the BAL of mice treated with vehicle. In vivo treatment with CH223191 did not alter the ability of macrophages to phagocytose *P. aeruginosa* but prevented the reduction in phagocytic activity of macrophages after treatment with kynurenine (Figure 5K). Overall, these data demonstrated that the inhibition of phagocytosis and killing of *P. aeruginosa* by kynurenine occurs in an AHR‐dependent manner. Therefore, heightened AHR activation by kynurenine during dysbiosis impairs phagocytosis of *P. aeruginosa* by leukocytes.

## 4 Discussion

The treatment of people infected with *P. aeruginosa* has become a major challenge, as this bacterium is becoming increasingly resistant to available antibiotics. Therefore, it is crucial to identify the host pathways that increase susceptibility to infection, as they could be potential candidates for therapies targeting the host. Alterations in the composition of the gut microbiota have been shown to disrupt the microbiota‐host relationship, leading to changes in the immune response and making the host more susceptible to pulmonary inflammatory and infectious diseases [30]. In this study, we have shown that: [1] antibiotic‐induced dysbiosis of the gut microbiota increases susceptibility to lung infection with *P. aeruginosa* in mice; [2] imbalances host tryptophan metabolism; [3] infection with *P. aeruginosa* induces an increase in kynurenine levels in the lung during eubiosis and dysbiosis; [4] increased IDO1 activity and AHR expression in dysbiotic infected mice; [5] IDO1 is important for regulating phagocytosis of *P. aeruginosa* during dysbiosis; [6] kynurenine impairs control of *P. aeruginosa* and [7] kynurenine‐associated impairment of phagocytosis is AHR‐dependent.

First, we have shown that intestinal dysbiosis induced by an antibiotic cocktail can alter the host gut microbiota and tryptophan metabolism. Several studies have shown that the intestinal microbiota can directly and indirectly affect host tryptophan metabolism. For example, germ‐free mice have high circulating tryptophan levels and low kynurenine and serotonin levels [21, 31]. Here, we observed that the use of an antibiotic cocktail could decrease the burden of bacteria important for tryptophan metabolism, such as enterobacteria and lactic acid‐producing bacteria, which could explain the increase in serum tryptophan levels. Furthermore, from the decrease in kynurenine levels in the serum of dysbiotic mice, we can infer a low systemic activity of the enzyme IDO1. In agreement with our results, previous studies have shown that intestinal epithelial cells express large amounts of the enzyme IDO1 and that this expression depends on the intestinal microbiota [21]. These processes could therefore contribute to the increase in tryptophan concentration observed in the sera of dysbiotic mice.

Importantly, we have shown that dysbiotic mice are more susceptible to *P. aeruginosa* infection. In dysbiotic mice, the burden of *P. aeruginosa* was increased in the BAL and the lungs. Similarly, other studies have shown that antibiotic‐induced changes in the gut microbiota are associated with an increase in *P. aeruginosa* load [23, 32]. The same profile has been observed with *Mycobacterium tuberculosis* . Mice receiving antibiotics were more susceptible to infection with *M. tuberculosis* and had an increased bacterial load in the lungs and spleen [33]. Therefore, alteration of the microbiota by antibiotics may lead to susceptibility to a broad spectrum of pulmonary pathogens. It is important to highlight that our model of antibiotic treatment differs significantly from the regimens in clinical use. Nevertheless, our results and results from other studies support the conclusion that antimicrobial therapy‐induced dysbiosis impairs host resistance to lung infection.

We also have shown that infected dysbiotic mice presented an impairment in neutrophil influx into the BAL and lung (as demonstrated by the histopathology analysis). Neutrophils are an essential component of the innate immune response to induce host resistance to *P. aeruginosa* infection since several studies have already demonstrated that the depletion of neutrophils or neutrophil products increases mortality upon *Pseudomonas aeruginosa* infection [34, 35, 36]. In this way, the decrease in neutrophil recruitment in dysbiotic mice correlates with a higher bacterial load in their lungs, which can allow the bacteria to reach the bloodstream, causing systemic infection, explaining the increased lethality in these mice. Additionally, there may be remote tissue injury in these mice that further contributes to their higher mortality rate.

In addition to inducing gut microbiota dysbiosis, our findings indicate that the use of the antimicrobial cocktail not only induces dysbiosis in gut microbiota but also alters the lung microbiota. The study of lung microbiota is relatively new, as the lower respiratory tract was considered to be sterile in healthy conditions for many decades. Although some studies have described the composition of lung microbiota, it remains unclear whether this microbiota can metabolise and influence the concentration of tryptophan metabolites. Although the antimicrobial cocktail is capable of altering the lung microbiota, the results from experiments involving exposure to streptomycin, an antimicrobial that does not affect the lung microbiota, suggest that dysbiosis of the intestinal microbiota appears to be responsible for the increased susceptibility of animals to infection by *P. aeruginosa* .

Intestinal dysbiosis is closely related to changes in tryptophan metabolites in the intestine, leading to dysregulation of the intestinal immune response [37]. Here we demonstrate that dysbiotic‐infected mice showed an increased Kyn:Trp ratio in the lungs. Tryptophan can be metabolised into kynurenine by two enzymes: IDO and tryptophan 2,3‐dioxygenase. In the lungs, the IDO1 enzyme is expressed by both lung epithelial cells and inflammatory cells, while TDO1 is expressed at lower levels primarily in lung fibroblasts [38, 39]. Additionally, we found a decrease in the Kyn:Trp ratio in the lungs of IDO1‐deficient mice after infection, suggesting that IDO1 is the major enzyme involved in kynurenine production in our model.

IDO1 plays an important role in modulating the inflammatory response and acts mainly as an immunosuppressant. These functions include the differentiation of CD4^+^ T cells into Treg cells and their activation, making this enzyme an important player in autoimmunity and antitumor immune response [40]. In the present study, however, we have shown that IDO1 plays a previously neglected role in controlling the innate immune response to bacterial infections during infection with *P. aeruginosa* . The enzyme IDO1 is expressed in dendritic cells and macrophages, and its expression is strongly regulated in inflammatory environments, as inflammatory mediators such as type I and II interferons, TNF and microbial components strongly induce its expression [40, 41]. Infection with influenza A virus and the bacteria *Chlamydia muridarium*, *Chlamydia pneumoniae* and *Mycobacterium tuberculosis* leads to an increase in IDO1 activity and consequently to an accumulation of kynurenine at the site of infection [20, 42, 43, 44]. Similarly, we have shown that infection with *P. aeruginosa* leads to an accumulation of kynurenine in the lung, demonstrating for the first time the increase in IDO1 enzyme activity during *P. aeruginosa* infection. Furthermore, our data show that the absence of the IDO1 enzyme enhances the control of bacterial load in the lung during dysbiosis, suggesting that the IDO1 enzyme and host tryptophan catabolism play an important role in the gut‐lung axis during dysbiosis.

In addition, we have shown that kynurenine can reduce the influx of neutrophils after infection with *P. aeruginosa*, leading to an uncontrolled bacterial load. Consistent with our results, kynurenine was shown to inhibit neutrophil recruitment in a model of infection with uropathogenic *Escherichia coli* [45]. Although tryptophan metabolites were previously shown to activate the AHR receptor, the absence of the AHR did not alter neutrophil influx in dysbiotic mice, suggesting that the inhibition of neutrophil influx by kynurenine is independent of AHR activation. Kynurenine is known to play an antioxidant role [46], and H_2_O_2_ is known to be important for neutrophil recruitment [47]. Therefore, kynurenine could directly inhibit neutrophil influx by inhibiting H_2_O_2_ production. In this context, studies by our group have shown that H_2_O_2_ can induce neutrophil apoptosis, so the late absence of H_2_O_2_ could favour neutrophil accumulation [48]. In addition, other metabolites of the kynurenine pathway, such as kynurenic acid, are involved in the activation of the GPR35 receptor and may play a role in neutrophil recruitment [49]. However, the role of GPR35 and other tryptophan‐derived metabolites will be investigated in further studies.

Surprisingly, we found that the absence of IDO1 is important for the control of phagocytosis of *P. aeruginosa* by alveolar macrophages in dysbiotic mice, which may explain the better control of bacterial load in these mice, even though they presented a smaller amount of neutrophils with reduced ROS production during infection. Alveolar macrophages are the first line of defence against lung infections by microorganisms, including *P. aeruginosa* [50]. The intestinal microbiota is essential for priming and activation of these cells and has a significant impact on their phagocytic capacity [51]. Thus, several studies have also shown that alterations of the intestinal microbiota by antibiotics significantly affect the phagocytosis of microorganisms by alveolar macrophages [32, 33, 52, 53]. Interestingly, our results showed that dysbiotic WT mice exhibited increased AHR expression, which was not the case in dysbiotic IDO1^−/−^ mice. Indeed, a recent study has shown that overexpression of IDO1 in intestinal epithelial cells leads to an increase in AHR expression and activation [54], leading us to hypothesise that the greater activity of IDO1 in dysbiotic mice is responsible for the greater kynurenine production and the increase in AHR expression and activation in leukocytes.

Furthermore, we have shown in vitro that IDO1 absence leads to greater phagocytosis and killing and that exposure to high concentrations of kynurenine negatively affects bacterial elimination by BMDM. In addition, we have shown that high concentrations of kynurenine, an AHR agonist, also impair the phagocytosis of *P. aeruginosa* by neutrophils. Finally, treatment with kynurenine reduced phagocytosis of *P. aeruginosa* in vivo in an AHR‐dependent manner. However, it remains unclear if the effects of kynurenine in phagocytosis and killing of *P. aeruginosa* are secondary to increased AHR expression or not. Because both assays involve short time exposure to kynurenine (30–120 min), we believe that the control of bacterial phagocytosis seems to involve direct AHR activation by kynurenine and does not depend on inducing enhanced AHR expression.

We also observed an increase in I3C and IAA in the lungs of antibiotic‐treated mice. Interestingly, these metabolites may be derived from tryptophan catabolism by the host enzyme IL4i1, an enzyme that is up‐regulated by inflammatory stimuli in immune cells leading to the production of AHR ligands [55]. It has been shown that IDO1 and IL4i1 may work simultaneously in tryptophan catabolism [55]. In this way, these metabolites can also play an important role in AHR activation in the studied system. The role played by these metabolites will be explored in further studies.

We have also shown that the AHR receptor is involved in the uncontrolled growth of bacteria in dysbiotic mice. The AHR receptor has been shown to play an important role during infection with *P. aeruginosa* once AHR is activated by *P. aeruginosa* ‐derived pigments and quorum molecules, resulting in improved control of bacterial load [56, 57]. However, in the present study, treatment with an AHR antagonist did not affect the control of *P. aeruginosa* in eubiotic mice. Overall, these data support our hypothesis that an increase in IDO1 activity and consequently kynurenine production and AHR activation is responsible for the decreased phagocytosis of *P. aeruginosa* and the increase in bacterial load in dysbiotic mice, revealing a mechanism involved in the control of bacterial phagocytosis in the lung by the gut microbiota.

Higher expression of CYP1A1, an enzyme induced by AHR activation, has been shown to result in lower phagocytosis of *Escherichia coli* [58]. Similarly, a study showed that the use of an AHR agonist can alter the expression of *Cyp1a1* and decrease the internalisation of *Listeria monocytogenes* into hepatocytes [59]. This suggests that kynurenine inhibits phagocytosis of *P. aeruginosa* by inducing the Cyp1 complex enzyme. In addition, *P. aeruginosa* can metabolise tryptophan to kynurenine, leading to the synthesis of anthranilate and quinolones, important quorum‐signalling molecules that regulate the virulence program of the pathogen [60]. Interestingly, a previous study has shown that kynurenine production by *P. aeruginosa* acts as an eliminator of ROS production, allowing the bacteria to evade killing by neutrophils [46], an excess of kynurenine in the host could not only influence the host's immune response but also make the environment more favourable for bacterial survival by promoting their virulence.

Host‐targeted therapy aims to intervene in various aspects of the host response. These include modifying host pathways that are essential for replication or contribute to the persistence of the pathogen in the cell, enhancing the host immune response, blocking pathways that are disrupted by the pathogen and lead to hyperinflammation, and modulating the host immune response that causes unbalanced responses [2]. Here, IDO1‐mediated kynurenine production and the AHR activation pathway were shown to impair *P. aeruginosa* infection by interfering with bacterial phagocytosis and preventing neutrophil recruitment to the infection site. Therefore, we propose the IDO1‐kynurenine‐AHR pathway as a potential target for host‐directed therapy aiming to modulate the host immune response in patients using broad‐spectrum antimicrobials and infected with *P. aeruginosa* .

## Author Contributions

Camila Bernardo de Brito, Mauro Martins Teixeira, Caio Tavares Fagundes and Daniele G. Souza created the study design and wrote the manuscript. Camila Bernardo de Brito, Raquel Duque do Nascimento Arifa, Rafael de Oliveira Bezerra, Carlos Eduardo Dias Igídio, Bárbara Maria de Amorim‐Santos, Anna Clara Paiva de Menezes Santos, Micheli Fagundes, Larissa Mendes Barbosa, João Paulo Pezzini Barbosa and Rafaela Ribeiro Álvares conducted the data acquisition. Larissa Marcely Gomes Cassiano, Markus Kohlhoff, Roney Santos Coimbra, Juliana Divina Almeida Raposo and Fernão Castro Braga performed quantitation of tryptophan metabolites. Alessandra M. Saliba provided *Pseudomonas aeruginosa* strains. Celso Martins Queiroz‐Junior performed the histopathology analysis. Camila Bernardo de Brito, Caio Tavares Fagundes and Daniele G. Souza analysed and interpreted data and performed the statistical analysis.

## Conflicts of Interest

The authors declare no conflicts of interest.

## Supporting information


**Figure S1.** The use of an antibiotic cocktail induces intestinal dysbiosis. Gate strategy for flow cytometry analysis: Ly6G^high^CD11b^high^F4/80^+/‐^ cells among CD45^+^ single events were considered neutrophils, while F4/80^high^CD11c^+^ cells among CD45^+^Ly6G^low^ single events were considered macrophages (A). C57/BL6 mice were exposed to 14 days of the antibiotic cocktail. At the end of the protocol, mice were euthanized and the faeces were harvested for analysis of the following bacterial groups: total aerobic bacteria (A), lactic acid producing bacteria (B), and enterobacteria (C). Also, was quantified indole production by faecal microbiota (D). Statistical analysis was performed using Student's *t*‐test. *p < 0.05 versus H_2_O. Experimental *N*: 5–8.
**Figure S2.** Antibiotic‐induced intestinal dysbiosis increases susceptibility of mice to PA103 and PAO1 infection. C57/BL6 mice were exposed to 14 days of the antibiotic cocktail. At the end of the protocol, mice were infected intranasally with 10^4^ CFU of the PA103 strain and were followed for 7 days to analyse the survival rate (A). Results are shown as a percentage of survival postinfection. Experimental *N* = 6 to 8. Statistical analysis to compare survival curves was performed using the Log‐rank test (Mantel‐cox) test. Also, 24 h after the protocol infection, mice were euthanized and BAL and lungs were harvested for the following analysis: bacterial load in BAL (B) and lung (C). C57/BL6 mice were treated with streptomycin (0.5 g/250 mL of water) or with the antibiotic cocktail in drinking water for 14 days. At the end of the protocol, mice were intranasally infected with 10^7^ CFU of the PAO1 strain. At 24 h after infection, mice were euthanized and BAL (D) and lungs (E) were collected for analysis of bacterial burden. Experimental *N*: 4–6. In B and C statistical analysis was performed using Student's *t*‐test. In D and E statistical analysis was performed with a one‐way test ANOVA followed by a Newman–Keuls post‐test *p < 0.05 versus H_2_O.
**Figure S3.** The use of an antibiotic cocktail induces intestinal dysbiosis in IDO1 deficient mice. C57/BL6 WT and IDO1^−/−^ mice were exposed to 14 days of the antibiotic cocktail. At the end of the protocol, mice were euthanized, and the faeces were harvested for analysis of the following bacterial groups: total aerobic bacteria (A) and lactic acid producing bacteria (B). *p < 0.05 versus H_2_O. Experimental *N*: 5.
**Figure S4.** Dysbiotic IDO1−/− mice do not present a decrease in inflammatory score and kynurenine and do not change cell viability and *P.aeruginosa* growth. C57/BL6 WT and IDO1^−/−^ mice were exposed to 14 days of the antibiotic cocktail. At the end of the protocol, mice were infected intranasally with 10^7^ CFU of the PAO1 strain and euthanized 24 h after infection, and lung collected to evaluate tissue injury using H&E (A). C57/BL6 mice were submitted to dysbiosis protocol and PAO1 strain infection. 2 h after infection, mice were euthanized, and the total number of leukocytes in BAL was determined (B). Alveolar macrophages from WT and IDO1^−/−^ mice were isolated and plated in 96‐well plates for the phagocytosis and killing assay to determine the CFU of phagocytosed *P. aeruginosa* (C) and the percentage of killing (D). *N* experimental: 5. Statistical analysis was performed using Student's *t*‐test. *p < 0.05 versus WT. BMDM cells from WT mice were treated with kynurenine at concentrations of 5 or 50 μM for 1 h. Experimental *N*: 5. After 1 h of treatment cell viability test by the MTT technique was performed (E). PAO1 strain as incubated with vehicle or kynurenine at concentrations of 5 or 50 μM. Then the bacterial growth was evaluated for 72 h (F).

## Data Availability

The authors confirm that the data supporting the findings of this study are available within the article and its Supporting Information.
