# Supplementary material for: Antibiotic‐Induced Dysbiosis of the Gut Microbiota Shifts Host Tryptophan Metabolism and Increases the Susceptibility of Mice to Pulmonary Infection With Pseudomonas aeruginosa
Source: Immunology. 2025 May 19;175(4):453–66. doi: 10.1111/imm.13932 (PMC12234421; doi:10.1111/imm.13932)
Supplement: Supplementary file 1 — Figure S1. The use of an antibiotic cocktail induces intestinal dysbiosis. Gate strategy for flow cytometry analysis: Ly6GhighCD11bhighF4/80+/‐ cells among CD45+ single events were considered neutrophils, while F4/80highCD11c+ cells among CD45+Ly6Glow single events were considered macrophages (A). C57/BL6 mice were exposed to 14 days of the antibiotic cocktail. At the end of the protocol, mice were euthanized and the faeces were harvested for analysis of the following bacterial groups: total aerobic bacteria (A), lactic acid producing bacteria (B), and enterobacteria (C). Also, was quantified indole production by faecal microbiota (D). Statistical analysis was performed using Student's t‐test. *p < 0.05 versus H2O. Experimental N: 5–8. Figure S2. Antibiotic‐induced intestinal dysbiosis increases susceptibility of mice to PA103 and PAO1 infection. C57/BL6 mice were exposed to 14 days of the antibiotic cocktail. At the end of the protocol, mice were infected intranasally with 104 CFU of the PA103 strain and were followed for 7 days to analyse the survival rate (A). Results are shown as a percentage of survival postinfection. Experimental N = 6 to 8. Statistical analysis to compare survival curves was performed using the Log‐rank test (Mantel‐cox) test. Also, 24 h after the protocol infection, mice were euthanized and BAL and lungs were harvested for the following analysis: bacterial load in BAL (B) and lung (C). C57/BL6 mice were treated with streptomycin (0.5 g/250 mL of water) or with the antibiotic cocktail in drinking water for 14 days. At the end of the protocol, mice were intranasally infected with 107 CFU of the PAO1 strain. At 24 h after infection, mice were euthanized and BAL (D) and lungs (E) were collected for analysis of bacterial burden. Experimental N: 4–6. In B and C statistical analysis was performed using Student's t‐test. In D and E statistical analysis was performed with a one‐way test ANOVA followed by a Newman–Keuls post‐test *p < 0.05 versus H2O. [file IMM-175-453-s001.docx]

**
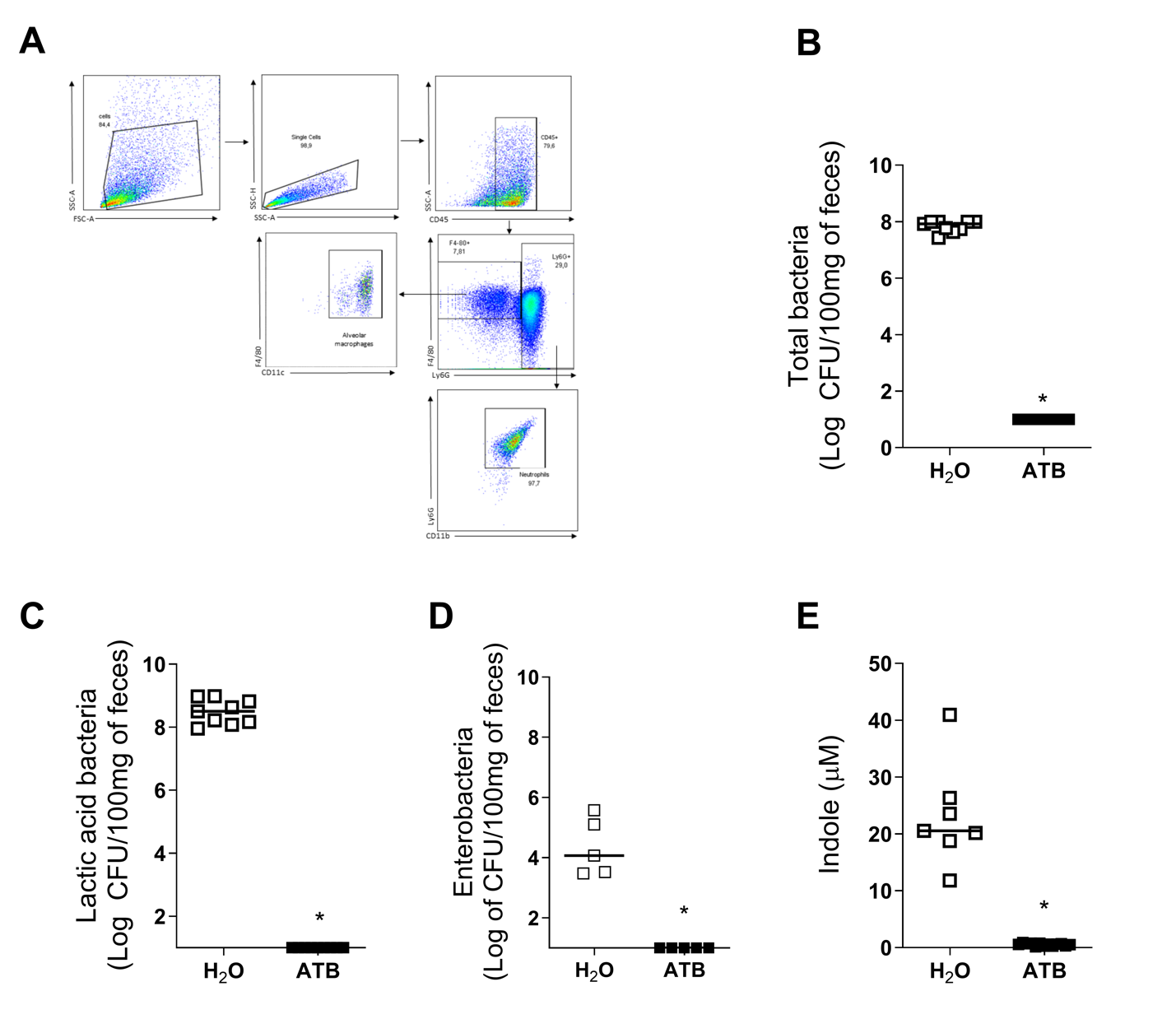
**

**Supplementary figure 1: The use of an antibiotic cocktail induces intestinal dysbiosis**. Gate strategy for flow cytometry analysis: Ly6G^high^CD11b^high^F4/80^+/-^ cells among CD45^+^ single events were considered neutrophils, while F4/80^high^CD11c^+^ cells among CD45^+^Ly6G^low^ single events were considered macrophages (A). C57/BL6 mice were exposed to 14 days of the antibiotic cocktail. At the end of the protocol, mice were euthanized and the feces were harvested for analysis of the following bacterial groups: total aerobic bacteria (A), lactic acid producing bacteria (B), and enterobacteria (C). Also, was quantified indole production by fecal microbiota (D). Statistical analysis was performed using Student's t-test. *P<0.05 vs H_2_O. Experimental N: 5 to 8.


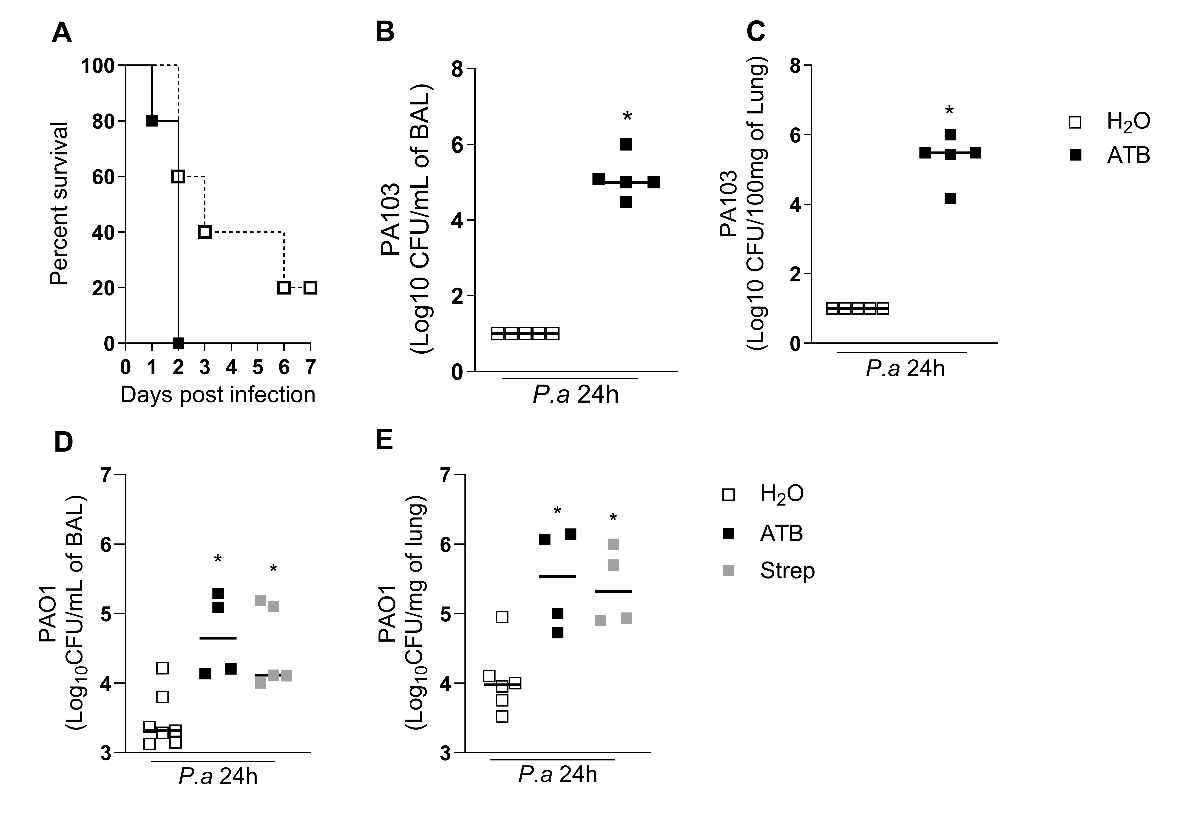


**Supplementary figure 2: Antibiotic-induced intestinal dysbiosis increases susceptibility of mice to PA103 and PAO1 infection.**  C57/BL6 mice were exposed to 14 days of the antibiotic cocktail. At the end of the protocol, mice were infected intranasally with 10^4^ CFU of the PA103 strain and were followed for 7 days to analyze the survival rate (A). Results are shown as a percentage of survival postinfection. Experimental N= 6 to 8. Statistical analysis to compare survival curves was performed using the Log-rank test (Mantel-cox) test. Also, 24h after the protocol infection, mice were euthanized and BAL and lungs were harvested for the following analysis: bacterial load in BAL (B) and lung (C). C57/BL6 mice were treated with streptomycin (0.5g/250mL of water) or with the antibiotic cocktail in drinking water for 14 days. At the end of the protocol, mice were intranasally infected with 10^7^ CFU of the PAO1 strain. At 24 hours after infection, mice were euthanized and BAL (D) and lungs (E) were collected for analysis of bacterial burden. Experimental N: 4-6. In B and C statistical analysis was performed using Student's t-test. In D and E statistical analysis was performed with a one-way test ANOVA followed by a Newman-Keuls post-test *P<0.05 vs H_2_O.


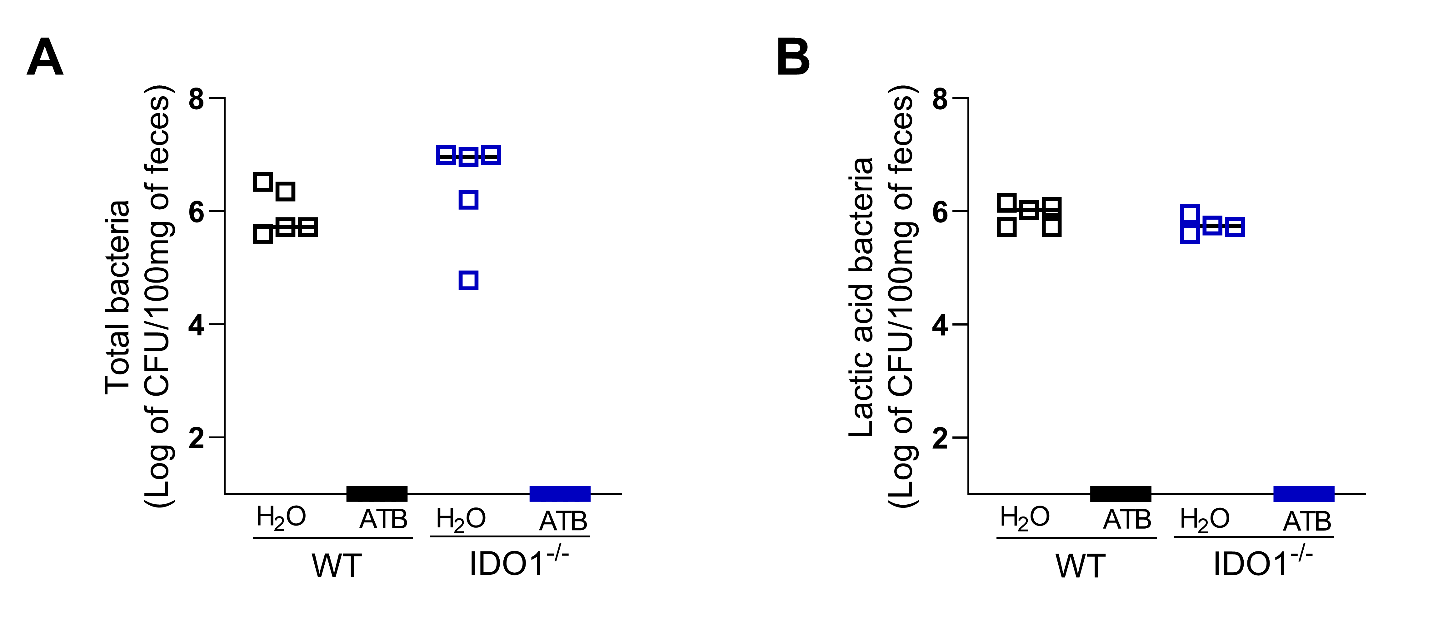


**Supplementary figure 3: The use of an antibiotic cocktail induces intestinal dysbiosis in IDO1 deficient mice.** C57/BL6 WT and IDO1^-/-^ mice were exposed to 14 days of the antibiotic cocktail. At the end of the protocol, mice were euthanized, and the feces were harvested for analysis of the following bacterial groups: total aerobic bacteria (A) and lactic acid producing bacteria (B). *P<0.05 vs H2O. Experimental N: 5.


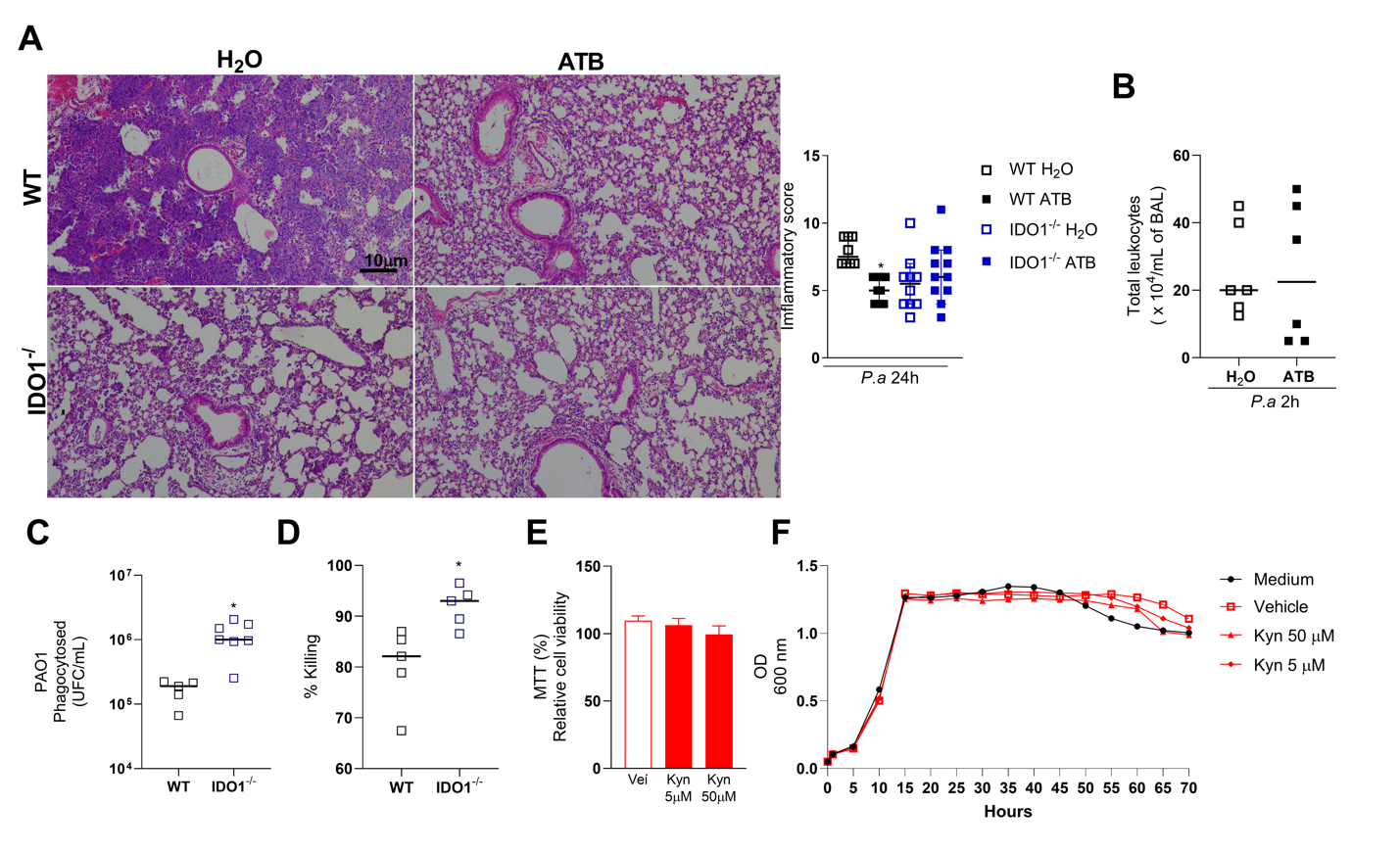


**Supplementary figure 4: Dysbiotic IDO1-/- mice do not present a decrease in inflammatory score and kynurenine and do not change cell viability and *P.aeruginosa* growth.** C57/BL6 WT and IDO1^-/-^ mice were exposed to 14 days of the antibiotic cocktail. At the end of the protocol, mice were infected intranasally with 10^7^ CFU of the PAO1 strain and euthanized 24h after infection, and lung collected to evaluate tissue injury using H&E (A). C57/BL6 mice were submitted to dysbiosis protocol and PAO1 strain infection. 2h after infection, mice were euthanized, and the total number of leukocytes in BAL was determined (B). Alveolar macrophages from WT and IDO1^-/-^ mice were isolated and plated in 96-well plates for the phagocytosis and killing assay to determine the CFU of phagocytosed *P. aeruginosa* (C) and the percentage of killing (D). N experimental: 5. Statistical analysis was performed using Student's t-test. *P<0.05 vs WT. BMDM cells from WT mice were treated with kynurenine at concentrations of 5 or 50µM for 1 hour. Experimental N: 5. After 1h of treatment cell viability test by the MTT technique was performed (E). PAO1 strain as incubated with vehicle or kynurenine at concentrations of 5 or 50µM. Then the bacterial growth was evaluated for 72h (F).
